# Why patterns of assortative mating are key to study sexual selection and how to measure them

**DOI:** 10.1007/s00265-015-2041-7

**Published:** 2015-11-27

**Authors:** Grant C. McDonald, Tommaso Pizzari

**Affiliations:** Department of Zoology, Edward Grey Institute, University of Oxford, Oxford, OX1 3PS UK

**Keywords:** Mating system, Sperm competition, Sexual selection, Sexual networks, Nestedness NODF, Newman’s assortativity, SCIC

## Abstract

The study of sexual selection is being revolutionised by the realisation that most populations exhibit some degree of polyandry, i.e. females mating with multiple males. Polyandry can drastically change the operation of sexual selection on males as it reduces the reproductive success that males derive by mating with different females, by forcing their ejaculates to compete for fertilisation after copulation (sperm competition). Variation in polyandry within a population means that the impact of polyandry can differ drastically across males, depending on the polyandry of their own mating partners. Because the patterns through which males share mates within a population may have strong repercussions for variation in male reproductive success, measuring such patterns is critical to study the operation of sexual selection. Several methods have been proposed to measure the pattern of mate sharing at the population level. Here, we develop a new method (sperm competition intensity correlation, SCIC) and compare its performance against two established methods (Newman’s assortativity and nestedness), using both idealised model populations and random simulated populations, across a range of biologically relevant population parameters: (i) population size, (ii) sex ratio and (iii) the ‘mating density’ of the population. We conclude that SCIC may be the most promising approach, as it is both internally consistent and robust across the parameter range. We discuss some important caveats and provide advice regarding the choice of method for future studies of sexual selection.

**Electronic supplementary material:**

The online version of this article (doi:10.1007/s00265-015-2041-7) contains supplementary material, which is available to authorized users.

## Introduction

Sexual selection is the selective process arising from variation in reproductive success due to intrasexual competition over access to mates and their gametes (Darwin 1871; Andersson 1994). Traditionally, competition over mates was viewed as the main source of intrasexual variation in reproductive success, and the strength of sexual selection on mating success was measured by the Bateman gradient, i.e. the slope of the univariate regression of total reproductive success, *T* over mating success, *M*:

1 $$ T(M)=\left({\beta}_M\times M\right)+\varepsilon $$

The Darwin-Bateman paradigm predicts that the male Bateman gradient (i.e. ${\beta}_{M_{\mathrm{males}}}$) is typically steeper than that of females, due to stronger precopulatory sexual selection on male mating success (Bateman 1948; Andersson 1994; Parker and Pizzari 2015). Less appreciated, however, are the profound implications that female mating success (*M*_females_) can have on the strength of sexual selection on males. When females are polyandrous (*M*_females_ > 1) such that the ejaculates of multiple males overlap at the time of fertilisation (Pizzari and Wedell 2013), the scope of male monopolisation is fundamentally changed. This is because, for males, mating is no longer a zero sum game as females become a shared resource, and males continue to compete after mating for the fertilisation of sets of ova, via sperm competition (Parker 1970) and under cryptic female choice (Childress and Hartl 1972; Thornhill 1983). Therefore, in polyandrous populations (i.e. female mating success can be greater than one), male reproductive success is not only contingent on the number of females mated and their fecundity but—crucially—also on the proportion of ova produced by his partners that a male manages to fertilise (Webster et al. 1995). Males that mate with the same female share the paternity of her ova, and the proportion of ova that a male fertilises is therefore a function of the intensity of sperm competition (sensu Parker 1998) faced by the male’s ejaculates across the females that he inseminates: the more intense the sperm competition, the lower the average share of paternity for each male (Shuster and Wade 2003; Parker and Pizzari 2010). In polyandrous populations, sperm competition intensity is therefore a key determinant of male reproductive success (Parker and Pizzari 2010; Collet et al. 2012; Kvarnemo and Simmons 2013; Parker and Birkhead 2013). Crucially, when individual females in a population vary in their polyandry (variance in *M*_females_ > 0), some males have the potential to suffer higher sperm competition intensity than others. The way in which sperm competition intensity is distributed across males dictates population-level patterns in sexual selection, determining both the total variation in male reproductive success and the strength of precopulatory sexual selection on mating success (i.e. ${\beta}_{M_{\mathrm{males}}}$). For example, consider a population where males with relatively high mating success tend to mate with the most polyandrous females and as a consequence face intense sperm competition. Despite the relatively high mating success of these males, their reproductive success is limited by the high polyandry of their partners, which reduces their paternity share. At the population level, such non-random mating patterns may weaken sexual selection on male mating success (i.e. ${\beta}_{M_{\mathrm{males}}}$), as further increases in mating success result in diminishing gains in fertilisation success (Sih et al. 2009; McDonald et al. 2013; McDonald and Pizzari 2014). Similarly, a negative relationship between male mating success and the mating success of his mates at the population level would strengthen ${\beta}_{M_{\mathrm{males}}}$. Understanding the operation of sexual selection therefore requires a measure of the relationship between the mating success of a male and the sperm competitive environment faced by his ejaculates, i.e. the mating success (i.e. polyandry) of his partners within a population (Sih et al. 2009; McDonald and Pizzari 2014).

**Fig. 1:**
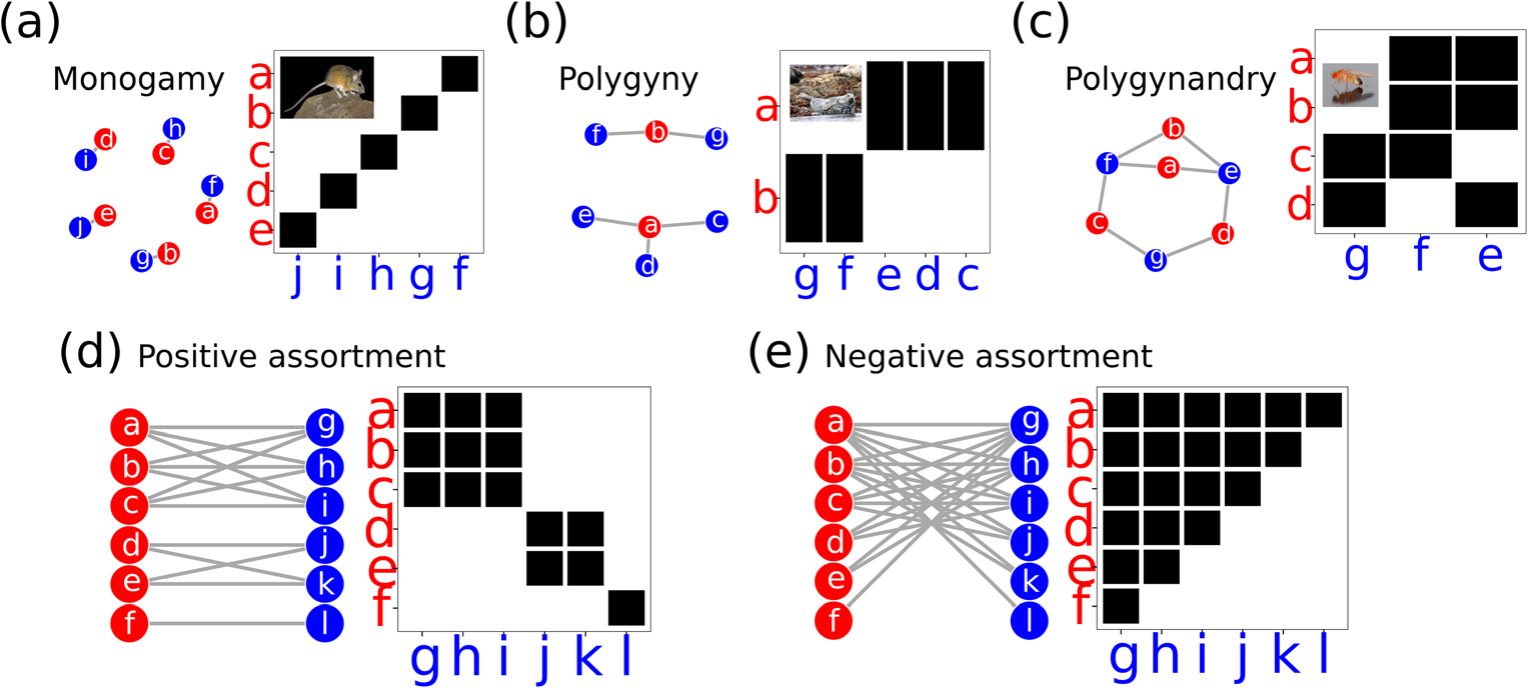
Each panel shows two visualisations of mating populations as sexual networks. Network visualisations show nodes representing individual males (*red*) or females (*blue*) and links (edges) represent copulations. Matrix representations show the same populations where males are *rows*, females are *columns* and *filled squares* represent copulations. **a** Monogamous population. **b** pPolygynyous population. **c** Polygynandrous population. **d** Example of positive mating assortativity (**e**) negative mating assortativity. Inset pictures courtesy of Wikimedia commons https://commons.wikimedia.org, from *left* to *right*: Californian mouse (*Peromyscus californicus*) by Whatiguana (Own work) [CC BY-SA 3.0 (http://creativecommons.org/licenses/by-sa/3.0) or GFDL (http://www.gnu.org/copyleft/fdl.html)], grey seal (*Halichoerus grypus*) by Steenbergs (Grey Seal On Farne Islands) [CC BY 2.0 (http://creativecommons.org/licenses/by/2.0)] and fruit fly (*Drosophila melanogaster*) by André Karwath aka Aka (Own work) [CC BY-SA 2.5 (http://creativecommons.org/licenses/by-sa/2.5)]

Borrowing techniques developed in both social science and ecological research focusing on food webs and mutualistic interactions, two such measures have recently been proposed: Newman’s assortativity (*r*) and nestedness metric based on overlap and decreasing fill (NODF) (Newman 2002; Almeida-Neto et al. 2008). Both methods utilise a network perspective of sexual interactions, where mating populations are described as a collection of nodes (males and females) that are connected by edges representing copulations (Fig. 1). Such sexual networks have been extensively explored in studies investigating the spread of sexually transmitted infections (Gupta et al. 1989; Liljeros et al. 2001), but their application to sexual selection begun only recently (McDonald et al. 2013; McDonald and Pizzari 2014; Inghilesi et al. 2015; Muniz et al. 2015). Mating patterns show a tremendous variety both between and within populations (Emlen and Oring 1977; Thornhill and Alcock 1983; Clutton-Brock 1989; Andersson 1994; Shuster and Wade 2003; Shuster 2009). The rapid surge in the availability of fine-grained behavioural data sets in combination with molecular parentage assignment (e.g. Preston et al. 2005; Rodríguez-Muñoz et al. 2010; Collet et al. 2012; Pélissié et al. 2014) is generating increasing scope for network approaches, and metrics such as Newman’s assortativity (*r*) and nestedness (NODF) are gaining prominence as tools to quantify mating patterns. Yet, despite this potential, we are aware of no analytical or quantitative evaluations of these methodological tools.

Here, we first review both, Newman’s assortativity (*r*) and nestedness (NODF), in the context of sexual networks and introduce a third measure that we term ‘sperm competition intensity correlation’ (SCIC). Secondly, we compare the performance of these three metrics in quantifying the assortativity of mating patterns based on mating success. Thirdly, we use randomly organised simulated populations to explore the sensitivity of these three metrics to each of three key axes of sexual selection variation: (i) population size, (ii) population adult sex ratio and (iii) the ‘mating density’ of the population. ‘Mating density’ measures the percentage of male–female pairs that copulate out of all possible heterosexual pairing combinations in a given population and thus reflects the saturation of a sexual network (i.e. the overall promiscuity of a population), independently of population size or sex ratio (see below). Furthermore, we examine how these three metrics (i.e. Newman’s *r*, NODF and SCIC) relate to each other across these three axes. Finally, in light of these results, we consider the application of these metrics to the study of sexual selection.

## Methods

### Sexual networks and measures of the mating structure

A sexual network is a collection of individuals (nodes) connected by links (edges) representing (inter- and/or intra-) sexual interactions, where the total number of links of a node is its degree (Croft et al. 2008; Wey et al. 2008; Sih et al. 2009; McDonald et al. 2013; Krause et al. 2014; McDonald and Pizzari 2014). In this study, we are interested in sperm competition so we focus on bipartite networks, that is networks with two sets of nodes, male and females, with edges between heterosexual mating partners and where an individual’s node degree represents its mating success. Conceptually, edges in these networks can either be directed or undirected. In an undirected network, when one male and one female are connected to each other, both are treated as having one link (male and female degree = 1). In a directed network, if one male and one female are connected, one individual (e.g. the male) is considered as having an outward link with that female (out-degree = 1, in-degree = 0) and the female is considered as having one inward link with that male (out-degree = 0, in-degree = 1). When females are polyandrous, their degree is greater than 1 and measures the intensity of sperm competition faced by their male partners (Parker 1970; Parker and Birkhead 2013). Below, we introduce three network metrics aimed at quantifying the relationship between a male’s mating success and that of his partners: Newman’s assortativity (*r*_Newman_), nestedness (NODF) and SCIC.

### Newman’s assortativity (*r*_Newman_)

Newman’s assortativity (*r*_Newman_) measures the assortativity of a network as the Pearson correlation coefficient between the trait values at either end of an edge (i.e. the link connecting a female to her mating partner), across all edges in the network (Newman 2002, 2003). The trait of interest in this context is node degree, which measures individual mating success (see above). Developed for unipartite networks (i.e. networks with one type of node, where edges can be potentially drawn between all individuals), *r*_Newman_ can be defined as:

2 $$ {r}_{\mathrm{Newman}}=\frac{{\displaystyle \sum }{j}_i{k}_i-{E}^{-1}{\displaystyle \sum }{j}_i{\displaystyle \sum }{k}_i}{\sqrt{\left[{\displaystyle \sum }{j}_i^2-{E}^{-1}{\left({\displaystyle \sum }{j}_i\right)}^2\right]\left[{\displaystyle \sum }{k}_i^2-{E}^{-1}{\left({\displaystyle \sum }{k}_i\right)}^2\right]}} $$

where *E* is the total number of edges in the network and *j*_*i*_ and *k*_*i*_ are node degrees of individuals *j* and *k* at either end of the *i*th edge. Therefore, *r*_Newman_ ranges from 1 (positive correlation, positive assortativity) to −1 (negative correlation, disassortativity). In a network of one monogamous mating pair, *E* equals 2 if we treat edges as undirected or 1 if we treat edges as directed. This is because in undirected networks, the edge is considered mutual and the correlation between trait values (in this case node degree) between linked nodes is therefore viewed from both directions (Newman 2003). To highlight this difference, consider the following example network with two males as rows and two females as columns, $$ \left[\begin{array}{cc}\hfill 1\hfill & \hfill 1\hfill \\ {}\hfill 1\hfill & \hfill 0\hfill \end{array}\right], $$ where 1 means that the pair copulated. The directed Newman’s assortativity, *r*_Newman(*D*)_, would treat this network as having three edges (*E* = 3), where the trait values (i.e. mating success) for the outgoing male links are $$ \left(\begin{array}{c}\hfill 2\hfill \\ {}\hfill 2\hfill \\ {}\hfill 1\hfill \end{array}\right) $$ and the trait values for the female inward links are $$ \left(\begin{array}{c}\hfill 2\hfill \\ {}\hfill 1\hfill \\ {}\hfill 2\hfill \end{array}\right) $$. Alternatively, Newman’s assortativity for undirected networks, *r*_Newman(*U*)_, treats each link as a mutual connection and so would treat this network as having six edges (*E* = 6) where the trait values at either end of each link are $$ \left(\begin{array}{c}\hfill \begin{array}{c}\hfill 2\hfill \\ {}\hfill 2\hfill \end{array}\hfill \\ {}\hfill \begin{array}{c}\hfill 1\hfill \\ {}\hfill 2\hfill \end{array}\hfill \\ {}\hfill \begin{array}{c}\hfill 1\hfill \\ {}\hfill 2\hfill \end{array}\hfill \end{array}\right) $$ and $$ \left(\begin{array}{c}\hfill \begin{array}{c}\hfill 2\hfill \\ {}\hfill 1\hfill \end{array}\hfill \\ {}\hfill \begin{array}{c}\hfill 2\hfill \\ {}\hfill 2\hfill \end{array}\hfill \\ {}\hfill \begin{array}{c}\hfill 2\hfill \\ {}\hfill 1\hfill \end{array}\hfill \end{array}\right). $$

This means that *r*_Newman(*U*)_ can yield both positive and negative values of assortativity and behaves in a way similar to *r*_Newman(*D*)_ when populations approach an even sex ratio, but the metrics can generate drastically divergent estimates of assortativity when sex ratios deviate from unity.

## Nestedness (NODF)

Nestedness is a concept originally borne from ecological research, designed to quantify patterns of species co-occurrences in metacommunities, where sites with lower species richness contain reduced subsets of those sites with higher species richness (Ulrich and Almeida-Neto 2012). This concept was later applied to ecological networks such as mutualistic plant-pollinator networks (Ulrich et al. 2009). Mutualistic plant-pollinator networks are nested when specialist plants (those with few connections) tend to connect to the most generalist pollinators (those with many connections) and specialist pollinators tend to connect to the most generalist plants (Bascompte et al. 2003; Bascompte and Jordano 2007; Ulrich et al. 2009). In the context of sexual networks, a population is nested when males with few mating partners (i.e. low mating success; *M*) tend to mate with the most polyandrous females (i.e. females with high *M*), and males with the highest mating success tend to include amongst their partners the least polyandrous females (i.e. females with low *M*). Nestedness is therefore specific to patterns of disassortativity and bipartite networks, i.e. negative assortment between male mating success and the mating success of their partners (McDonald and Pizzari 2014) and is perhaps best visualised by using the matrix representation of sexual networks (Fig. 1e). Accordingly, the most widely used methods to calculate nestedness is NODF (Almeida-Neto et al. 2008; Strona and Fattorini 2014), which measures nestedness based on the rules of percentage overlap (PO) and decreasing fill (DF) between pairs of rows and pairs of columns of such matrix and quantifies the extent to which the mating partners of individuals with low degree form subsets of the mating partners of those individuals with a higher degree (McDonald and Pizzari 2014). To calculate nestedness as outlined by Almeida-Neto et al. (2008) for sexual networks, we first order the mating population matrix of left to right (females) and top to bottom (males) in order of decreasing mating success. Then, for every pair of *i* and *j* rows (males), we calculate two values, DF (decreasing fill) and PO (percentage overlap). To calculate DF, we ask whether the upper row in the pair (row_*i*_) has a higher mating success (*M*) than the lower row (row_*j*_). If *M*_*j*_ < *M*_*i*_, then DF_*ij*_ = 100 and if *M*_*j*_ ≥ *M*_*i*_ then DF_*ij*_ = 0. The PO of pairs of rows is calculated as the percentage of row_*j*_’s mating partners shared with row_*i*_. The same process is calculated for all pairs of columns (females), where the *i*th female is the leftmost and the *j*th female is the female to its right. For all pairs of rows and columns, we then calculate thier individual nestedness (*N*_*ij*_) as:

$$ \mathrm{If}\;D{F}_{ij}=0,\ \mathrm{then}\;{N}_{ij}=0 $$

$$ \mathrm{If}\kern0.5em D{F}_{ij}=100,\;\mathrm{then}\ {N}_{ij}=P{O}_{ij} $$

For example, consider the top male rows in Fig. 1e. The top male has six mates and the second male has five mates, therefore *DF*_*ij*_ = 100. The second male also shares all of his females with the top male, so the percentage overlap PO_*ij*_ = 100 and *N*_*ij*_ = 100. We can then measure the nestedness of the whole network as:

3 $$ \mathrm{NODF}=\frac{{\displaystyle \sum }{N}_{ij}}{\left[\frac{f\left(f-1\right)}{2}\right]+\left[\frac{m\left(m-1\right)}{2}\right]} $$

where *f* and *m* are the numbers of males and females, respectively, and NODF ranges between 100 (perfect nestedness) and 0 (no nestedness) (Almeida-Neto et al. 2008).

## SCIC

**Fig. 2:**
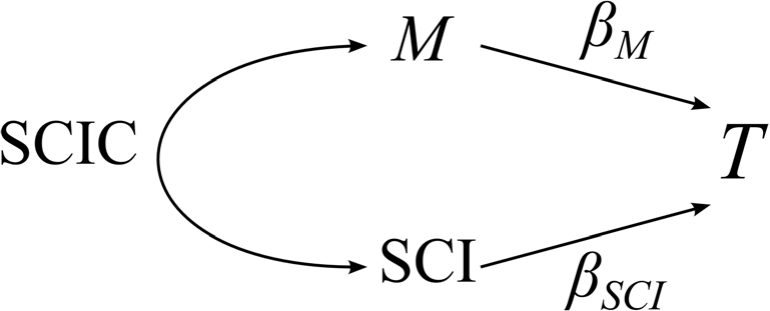
Path diagram showing the relationship between reproductive success (T), mating success (M), sperm competition intensity (SCI) and the relationship between the two (SCIC)

The aim of quantifying population-level patterns in assortative mating by mating success is to understand its influence on the strength of sexual selection on male mating success (i. e. *β*_*M*_). When females are polyandrous, males will be forced to sperm compete and, all else being equal, the reproductive success of a focal male is inversely proportional to the number of sperm competitors with which he competes (i.e. his ‘sperm competitive intensity’, SCI). The sperm competition intensity suffered by a male can be characterised by the harmonic mean of a male’s partners mating success (Shuster and Wade 2003; Wade and Shuster 2005). For example, consider a male that mates with two females; one of which does not remate, whilst the other copulates with two other males. The sperm of this male does not face sperm competition within the first female. For the second female however, the sperm of the focal male must compete with the sperm of two other males. Assuming the simplest null model of sperm competition (i.e. fair raffle and similar ejaculate size and fertilising efficiency across competitors), the focal male’s share of paternity for this female is $\frac{1}{3}$ (Shuster and Wade 2003). The average share of paternity of the focal male across the two partners is then $\frac{1}{2} \times \left(\frac{1}{1}+\frac{1}{3}\right) = 0.667$ where $\frac{1}{0.667}$ is the harmonic mean number of sperm competitors faced by the focal male (i.e. $\frac{1}{0.667} = 1.499$ competitor males). This can be calculated for the *i*th male simply as:

4 $$ {\mathrm{SCI}}_i=\frac{1}{\frac{1}{M_i}\left({\displaystyle {\sum}_j^M}\frac{1}{k_j}\right)} $$

where *k*_*j*_ is the mating success (degree) of the *j*th female mated with the *i*th male, and *M* is the total number of mating partners of the *i*th male (i.e. his mating success). This parameter can then be added to our regression model in Eq. 1 as:

5 $$ {T}_i=\left({\beta}_{M\cdot \mathrm{S}\mathrm{C}\mathrm{I}}\times {M}_i\right)+\left({\beta}_{\mathrm{SCI}\cdot M}\times {\mathrm{SCI}}_i\right)+\varepsilon $$

where *β*_*M* ⋅ SCI_ represents the male Bateman gradient controlling for variation in SCI. Crucially, the way in which the univariate Bateman gradient, *β*_*M*_, is affected by variation in sperm competition intensity across males is determined by the slope of the partial regression of mating success on the mean sperm competition intensity faced by a male (i.e. *β*_SCI ⋅ *M*_) and the correlation between *β*_*M* ⋅ SCI_ and *β*_SCI ⋅ *M*_ (i.e. SCIC, Fig. 2) as:

6 $$ {\beta}_M={\beta}_{M\cdot \mathrm{S}\mathrm{C}\mathrm{I}}+\left(\mathrm{SCIC}\times {\beta}_{\mathrm{SCI}\cdot M}\right) $$

In general, theory predicts that *β*_SCI ⋅ *M*_ will be negative as increased sperm competition decreases a male’s reproductive success (Shuster and Wade 2003). Therefore, when SCIC is positive, we predict a reduction in male Bateman gradients, whereas a negative SCIC will steepen the slope of the male Bateman gradient (Wolf et al. 1999). Typically, Bateman gradients are standardised by dividing reproductive success and mating success by their respective means (Jones 2009). In this fashion, we can also standardise SCI by its population mean and provide a standardised slope for SCIC that facilitates comparisons across populations. If instead, *M*_males_ was standardised by subtracting the population mean and dividing by its standard deviation, as for selection gradients on phenotypic traits (Lande and Arnold 1983; Wolf et al. 1999), SCIC could be calculated as the Pearson product correlation coefficient between SCI and *M*_males_. We provide R code to calculate SCIC as supplementary material (R Core Team 2014).

## Model populations

We develop model populations displaying a range of mating systems to test the performance of all the metrics (i.e. *r*_Newman(*U*)_, *r*_Newman(*D*)_, NODF and SCIC) in identifying mating success assortativity. Research has highlighted a tremendous variety both between and within populations (Emlen and Oring 1977; Thornhill and Alcock 1983; Clutton-Brock 1989; Andersson 1994; Shuster and Wade 2003; Shuster 2009). Variation in mating patterns (the mating topology) across populations can be decomposed into three main axes: (i) individual variation in male mating success (i.e. level of polygyny, var(*M*_males_)), (ii) individual variation in female mating success (i.e. level of polyandry, var(*M*_females_)) and (iii) the average level of polygyny and polyandry of the entire population (i.e. ${\overline{M}}_{\mathrm{males}}$_and_${\overline{M}}_{\mathrm{females}}$, respectively). In strictly monogamous populations with unitary sex ratio, ${\overline{M}}_{\mathrm{males}}={\overline{M}}_{\mathrm{females}}=1,$ and there is no intrasexual variation in either male or female mating success (Fig. 1a). On the other hand, in populations with unitary sex ratio where the mating success of one sex (but not the other) exceeds 1, there is variation in mating success amongst members of that sex but no variation in mating success in the opposite sex (Fig. 1b). For sperm competition to occur, populations must exhibit some level of polyandry (i.e. ${\overline{M}}_{\mathrm{females}}>1;$ Pizzari and Wedell 2013). Furthermore, for males and females to mate assortatively by mating success, there must also be some variation in both male and female mating success (var(*M*_males_) *>* 0, var(*M*_females_) > 0, not including individuals that do not mate at all). Any metric measuring the assortativity of mating partners based on mating success should therefore only provide non-zero results when all three conditions are met, namely ${\overline{M}}_{\mathrm{females}}>1,$ var(*M*_males_) > 0 and var(*M*_females_) > 0.

**Fig. 3:**
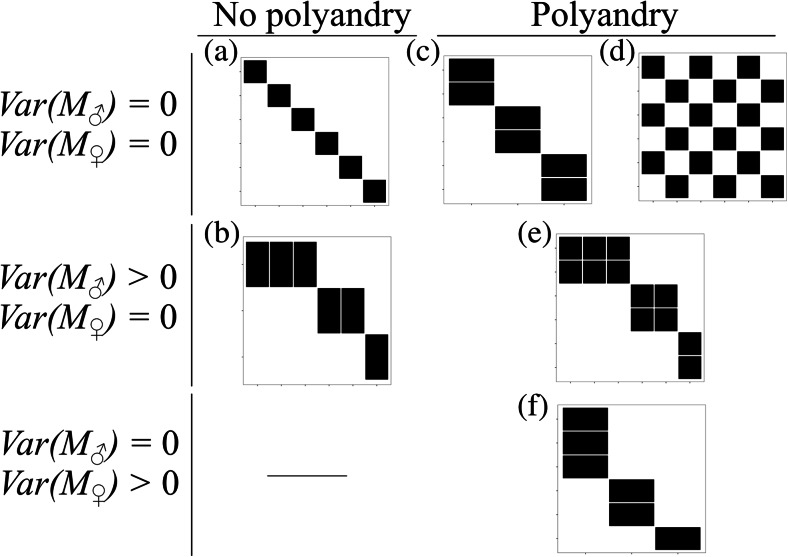
Model mating populations that show either variation in male or female mating success (Var(M)) and polyandry, but no population displays all three conditions simultaneously and so cannot demonstrate assortative/disassortative mating topologies

None of the developed model populations in Fig. 3 display all three conditions and thus are not expected to generate assortative mating by *M* different from zero. On the other hand, the model populations in Fig. 4 meet all three conditions for assortative mating by *M*. These populations represent different ‘mating densities’ (i.e. the proportion of possible mating pairs realised of all heterosexual pairing combinations possible in a given population) and display either positive or negative mating assortativity by *M*.

## Simulated populations

When measuring the relationship between male mating success and that of their partners, any useful metric should allow the estimation of such assortativity across a wide range of population parameters but should not be confounded by other naturally varying population parameters. Here, we test the performance of all metrics, *r*_Newman(*U*)_ and *r*_Newman(*D*)_, NODF and SCIC on randomly simulated mating populations along three fundamental gradients of variation in mating systems, namely (i) population size, (ii) sex ratio and (iii) ‘mating density’. We do so by varying each axis separately one at a time as outline below.

1. *Population size*: We generated populations that differ in the total number of individuals, keeping their ‘mating density’ (i.e. realised proportion of all possible mating combinations) and sex ratio constant. To do so, we created 1,000 populations of each of three sizes: 6 males and 6 females (6 × 6), 20 males and 20 females (20 × 20) or 80 males and 80 females (80 × 80), totaling 3,000 populations. We simulated mating by randomly connecting males and females until 50 % of the total possible mating pairs was realised ensuring that every individual mated at least once. Of the 1,000 populations with 6 males and 6 females (6 × 6), ten populations had zero variation in female mating success and four populations had zero variation in male mating success, so no value could be calculated for *r*_Newman(*D*)_ in the former and SCIC in the latter case. These populations were removed from analyses that included these two metrics, respectively.
2. *Sex ratio*: We created 1,000 populations with 10 males and 50 females (1:5), 30 males and 30 females (1:1) or 50 males and 10 females (5:1) and simulated mating by randomly connecting males and females until 50 % of the total possible mating pairs was realised ensuring that every individual mated at least once. Each population therefore had the same population size and *‘*mating density’.
3. *Mating density*: We measured the ‘mating density’ of a population as the percentage of male–female pairs that mated out of the total possible combinations of heterosexual mating pairs (i.e. for a population of 10 males and 10 females, there are 100 possible mating pairs and if 50 of those pairs actually mated, the population would have a ‘mating density’ of 50 % or a density of 0.5 in network nomenclature). ‘Mating density’ therefore gives an indication of how well connected sexual networks are that is independent of population size or sex ratio. This parameter is important as many network analysis measures are strongly affected on the overall density of networks (Croft et al. 2008). We simulated populations that differed in the realised proportion of all possible mating combinations (‘mating density’) but not in population size or sex ratio. To do so, we created 1,000 populations, all with 20 males and 20 females (i.e. same population size and sex ratio), where either 25, 50 or 75 % of possible pairs mated. We simulated mating by randomly connecting males and females until the target ‘mating density’ was achieved, ensuring that every individual mated at least once.

To assess the independence of assortative mating measures across each level of population size, sex ratio and *mating density*, we report the Spearman rank correlation coefficient between all assortative mating measures: *r*_Newman(*U*)_, *r*_Newman(*D*)_, NODF and SCIC, with all population parameters. To explore whether each measure is likely to capture similar information, we also calculated the Pearson product moment correlation coefficient between each assortative mating measures at each level of population size, sex ratio and *mating density*. All analyses were conducted using R statistical software (R Core Team 2014). As this study is entirely based on in silico simulations rather than empirical data, no blinded protocol was required.

## Results

### Model populations

We first assessed the consistency of each measure of assortative mating (*r*_Newman(*U*)_, *r*_Newman(*D*)_, NODF and SCIC) by applying all measures to the six model populations presented in Fig. 3. No population in Fig. 3 satisfies the criteria required for a correlation between male mating success and the mating success of his partners (i.e. polyandry and variation in both male and female mating success), and thus we should expect measures of assortative mating to return zero or no value for all six populations. As expected, all metrics returned zero or no value for all populations with the exception of *r*_Newman(*U*)_ that returned negative values for four populations (Fig. 3b, c, e and f and Table 1). This is because *r*_Newman(*U*)_ was originally intended for unipartite networks (networks with one type of node, e.g. all males). Therefore, although there is no variation in mating success within one sex (which would preclude the calculation of a correlation coefficient), *r*_Newman(*U*)_ instead correlates the mating success (degree) of individuals at either end of an edge (i.e. effectively counting each copulation twice from both the male and female end of an edge). Therefore, although there is no variation in degree within a sex, *r*_Newman(*U*)_ utilises variation between sexes when calculating the correlation between male and female mating success.

**Table 1:** Results for all assortativity metrics for model populations in Figs. 2 and 3

Model population	SCIC	NODF	*r*_Newman(*U*)_	*r*_Newman(*D*)_	Overall assortative mating pattern
Figure 3a	–	0.000	–	–	None
Figure 3b	0.000	0.000	−0.615	–	None
Figure 3c	–	0.000	−1.000	–	None
Figure 3d	–	0.000	–	–	None
Figure 3e	0.000	0.000	−0.091	–	None
Figure 3f	–	0.000	−0.615	–	None
Figure 4a	–0.385	0.000	−1.000	−1.000	Negative
Figure 4b	–0.342	33.333	−0.833	−0.833	Negative
Figure 4c	–0.555	100.000	−0.500	−0.500	Negative
Figure 4d	–0.193	33.333	−0.167	−0.167	Negative
Figure 4e	1.000	0.000	1.000	1.000	Positive
Figure 4f	0.515	20.000	0.550	0.550	Positive

– represents when no result is estimated by the method or is undefined (i.e. NA or NaN)

We next assessed the sensitivity of all metrics to detect positive or negative assortativity by mating success across a range of mating structures in idealised populations. All metrics provided qualitatively logical values, consistent with expectations across all matrices, with the exception of NODF that returned zero values for two populations (Table 1 and Fig. 4a, e). This inconsistency arises for two reasons: firstly, because despite the apparent negative correlation between male mating success and the mating success of their partners in the population in Fig. 4a, this population does not show both decreasing fill *and* overlap in the mating partners between any males. Secondly, the population shown in Fig. 4e is a completely positively assorted, and NODF is a measure specific to negative assortment (i.e. disassortativity) and thus identifies this population as zero nestedness. Therefore, only two parameters *r*_Newman(*D*)_ and SCIC provided consistent results across all model populations (Table 1).

## Simulated populations

1. *Population size*: All assortative mating measures were moderately correlated with population size, with the exception of NODF, which was decoupled from population size (Fig. 5a, d, g and j), confirming the result for NODF presented in Almeida-Neto et al. (2008). The correlations of *r*_Newman(*U*)_, *r*_Newman(*D*)_ and SCIC with population size tended towards zero as populations became larger. This observation is significant because it reveals that negative assortativity can emerge as a property of random mating in very small groups or populations, whereas deviations from 0 in larger populations more likely require a biological explanation (e.g. behavioural strategies).

**Fig. 4:**
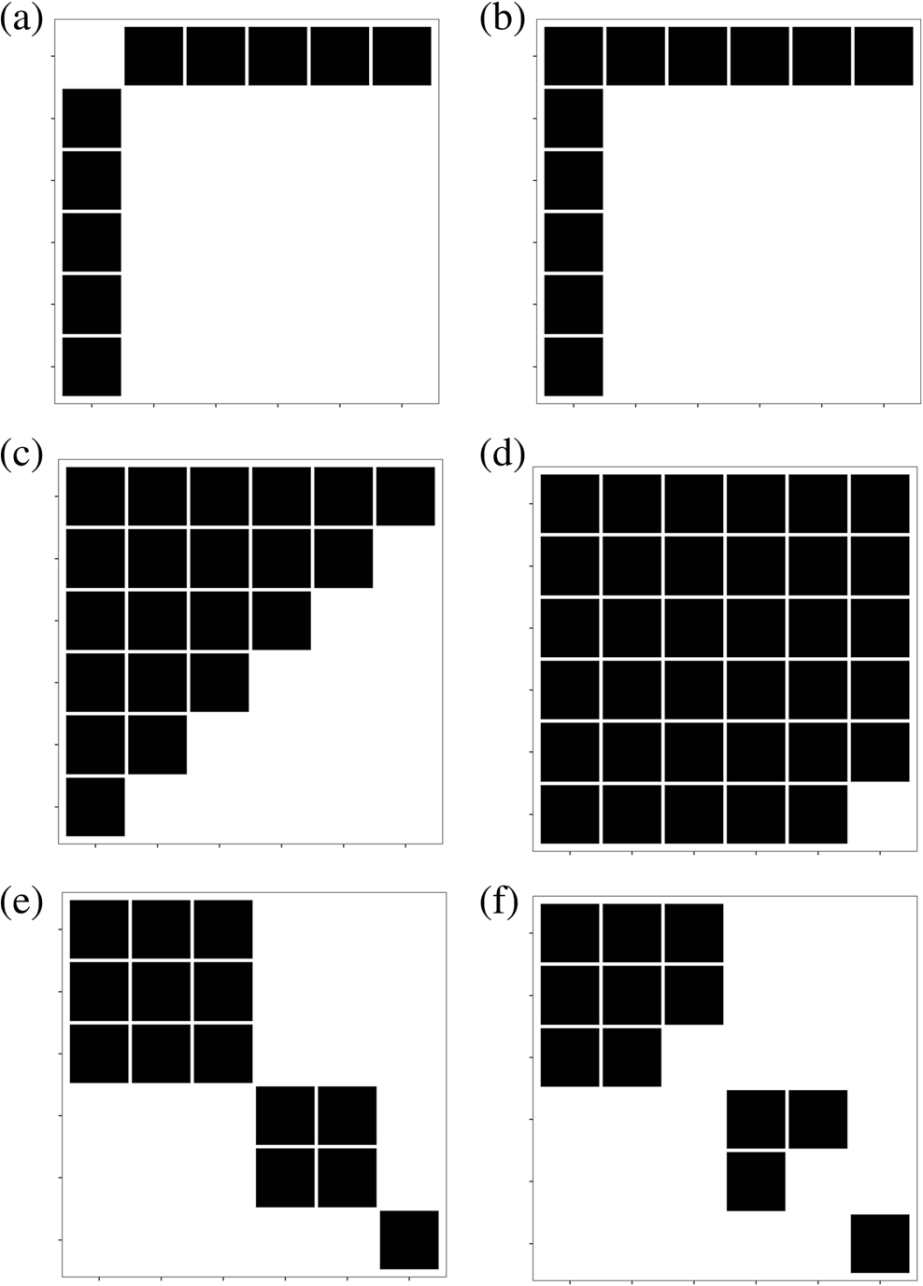
Model mating populations that simultaneously show polyandry and variation in male and female mating success (Var(M)) and so can demonstrate assortative/disassortative mating topologies. a Minimal ‘mating density’, strongly disassortative (e.g. alternative reproductive tactics). b Strongly disassortative (e.g. alternative reproductive tactics). c High ‘mating density’ and disassortative. d Maximal ‘mating density’ and disassortative. e High positive assortativity. f Imperfect positive assortativity with some disassortativity
2. *Sex ratio*: All assortative mating measures were very weakly or weakly correlated with population sex ratio (Fig. 5b, e, h and k). The measure *r*_Newman(*U*)_, however, showed a clear strong, non-monotonic relationship with sex ratio (Fig. 5b). When sex ratios were skewed in either direction, *r*_Newman(*U*)_ tended to be strongly negative. This is because uneven sex ratios resulted in one sex having a higher mean mating success than the other sex, and *r*_Newman(*U*)_ reflects the tendency for one sex with high mating success to mate with the other sex, which necessarily has a lower mean mating success.
3. *Mating density*: All assortative mating measures were very weakly correlated with the ‘mating density’ of populations (Fig. 5c, f and i), with the exception of NODF which was very strongly positively correlated with ‘mating density’ (Fig. 5l), confirming previous results for NODF (Almeida-Neto et al. 2008).
4. Correlations between assortative mating measures. Overall, for population size and ‘mating density’ simulations *r*_Newman(*U*)_, *r*_Newman(*D*)_ and SCIC tended to strongly positively correlate with each other (Fig. 6). Similarly, because increasing values of NODF suggest more negative assortment, all three of these measures tended to correlate negatively with NODF, although this correlation was weaker (Fig. 6). These results suggest that over most parameter ranges, these metrics capture similar information, although the correlations with NODF were much weaker, potentially because NODF specifically measures negative assortment. For sex ratio simulations, correlations involving *r*_Newman(*U*)_ showed a marked pattern across different sex ratios (Fig. 6). When populations diverged from an equal sex ratio, correlations between *r*_Newman(*U*)_ and all other measures reversed direction and weakened, reflecting the tendency for *r*_Newman(*U*)_ to identify increasingly negative assortativity values when sex ratios are biased in either direction. Overall, *r*_Newman(*D*)_ and SCIC were consistently and strongly correlated with each other across all parameter ranges (Fig. 6).

**Fig. 5:**
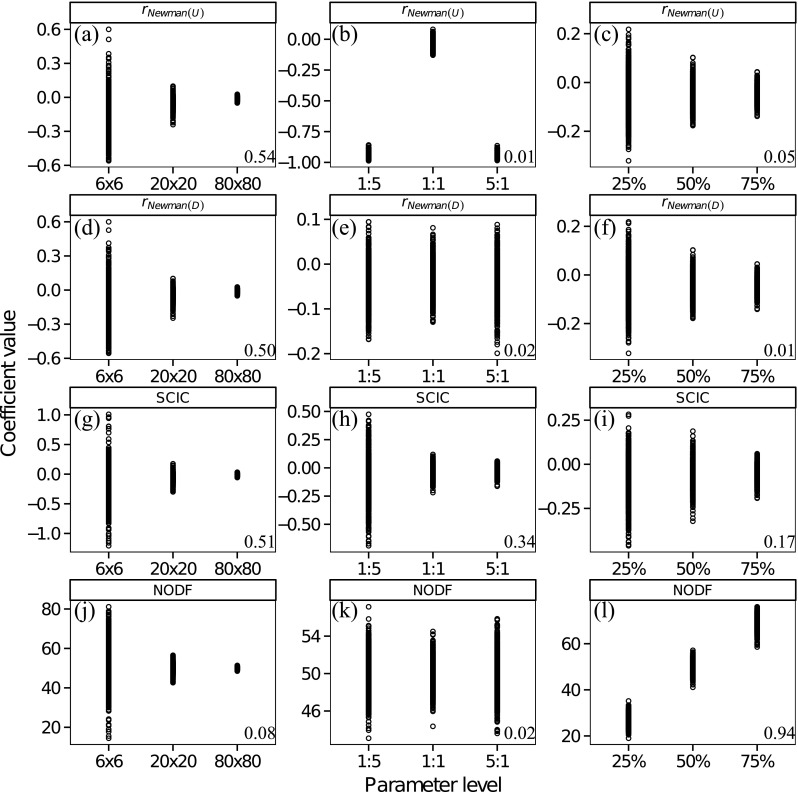
The relationship between measures of assortative mating structure (rNewman(U) (a–c, rNewman(D) (d–f), SCIC (g–i) and NODF (j–l) and population level parameters (population size (a, d, g, j)), sex ratio (males/females) (b, e, h, k) and ‘mating density’ (c, f, i, l) for randomly simulated mating populations. Numbers on the bottom right are the Spearman rank correlation coefficients

## Discussion

Multiple mating by females (polyandry) results in variance in male paternity share, creating opportunity for postcopulatory sexual selection via sperm competition and cryptic female choice (Parker 1970; Childress and Hartl 1972; Thornhill 1983). Consequently, in polyandrous populations, males can increase reproductive success by both mating with more females and/or increasing their paternity share (Alcock 1994; Alonzo and Warner 2000; Parker and Birkhead 2013), for example by maintaining exclusive access to mating partners by curtailing their polyandry (e.g. through mate guarding). Sexual selection on male mating success $\left({\beta}_{M_{\mathrm{males}}}\right)$ is stronger when males who mate with many females also enjoy a higher exclusivity of mating. Conversely, in populations with positive assortativity between male and female mating success, males with high mating success also face more intense sperm competition. This weakens selection on male mating success $\left({\beta}_{M_{\mathrm{males}}}\right)$ because increasing mating success results in decreasing returns in terms of reproductive success, due to reduced paternity share. Therefore, understanding inter-population variation in sexual selection hinges on characterising population-level patterns of the association between male mating success and the mating success of his partners (assortative mating by *M*).

In this work, we outlined multiple approaches to measures of the relationship between male mating success and the mating success of his partners, namely Newman’s assortativity for undirected and directed networks (*r*_Newman(*U*)_ and *r*_Newman(*D*)_) and nestedness (NODF) and introduced a third quantitative measure, the SCIC. We then used (i) idealised model populations to assess the logical performance of these measures in capturing assortative mating by mating success and (ii) simulated random mating populations to test the dependency of these measures on three main population parameters, namely population size, sex ratio and the ‘mating density’ of the population.

Our model populations allowed us to test the performance of all measures of assortative mating in situations where there should be zero assortativity or where there should be qualitatively positive or negative assortativity. Our results highlighted limitations in two measures, NODF and *r*_Newman(*U*)_ as both measures either indicate zero assortative mating by mating success when there should positive or negative assortativity, or alternatively indicated assortative mating when there should be none. Instead, *r*_Newman(*D*)_ and SCIC showed either no assortativity or were undefined when there should be zero assortative mating and qualitatively consistent results when there should be non-zero assortative mating.

Our simulations allowed us to test for the dependency of all assortative mating measures on population parameters expected to vary in nature, namely (i) population size, (ii) sex ratio and (iii) the ‘mating density’ of the population. Our results show that most measures were often not strongly associated with these population parameters. However, SCIC and *r*_Newman(*U*)_ showed a bias towards negative values at small populations. When populations are very small, those males that mate with many females will tend to on average compete with males who have lower mating success, purely as a result of small, restricted populations. Whether such patterns can be explained by random processes alone may be investigated through the use of randomised null models (Ulrich and Gotelli 2007). However, in such small populations, even small deviations from random may have a large effect of the distribution of male reproductive success and a first step should be to explore the link between measures of assortative mating and the operation of sexual selection. In two cases, we identified strong relationships between population parameters and measures of assortativity. In our sex ratio simulation, *r*_Newman(*U*)_ showed a strong non-monotonic relationship across different sex ratios. This is because, when sex ratios are skewed, the mean mating success of one sex is higher than the other resulting in negative values of *r*_Newman(*U*)_. For ‘mating density’ simulations, we demonstrated that NODF is strongly positively correlated with ‘mating density’, similar to previously published studies (Almeida-Neto et al. 2008). This relationship could be overcome by z-transforming NODF values via simulated distributions of observed mating networks controlling for ‘mating density’ (Almeida-Neto et al. 2008). However, the choice of null models used to simulate matrices may not be straightforward and would change the interpretation of empirical NODF values, to a deviation from randomness (Ulrich and Gotelli 2007; Strona and Fattorini 2014).

Overall, the two measures that were most consistent across all population parameters were *r*_Newman(*D*)_ and SCIC. Both measures correlated strongly and positively with each across all population parameters, suggesting they are likely to capture similar variation. However, arguably only SCIC has a clear relationship to sexual selection theory. This is because unlike *r*_Newman(*D*)_, SCIC can be directly included in the partitioning of the Bateman gradient into those components that reflect the independent effect of mating success and the independent effect of sperm competition on male reproductive success. Furthermore, SCIC can be used directly to quantify the contribution of polyandry and assortativity in mating patterns to Bateman gradients, by multiplying SCIC and the partial regression coefficient of the sperm competition intensity faced by a male (*β*_SCI ⋅ *M*_) (Fig. 2, Eq. 6).

**Fig. 6:**
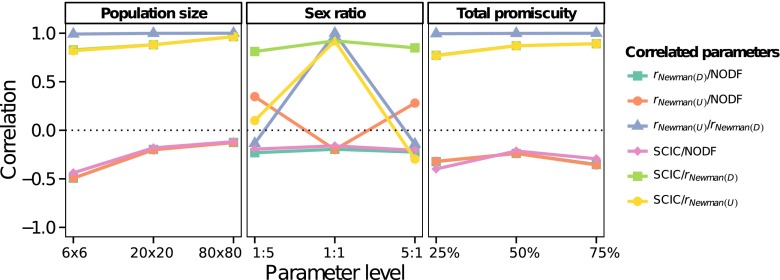
Plots show the Pearson product correlation coefficient between measures of assortative mating structure (rNewman(U), rNewman(D), SCIC and NODF) across all levels of population level parameters (population size, sex ratio (males/females) and ‘mating density) for randomly simulated mating populations

An important caveat is that our approach calculates SCI assuming that each mating has the same competitive value (equality of mating). This assumption may generate simplistic expectations in terms of variation in paternity share. Variation in male traits such as ejaculate size, factors that load the sperm competition raffle (e.g. mating order) and mechanisms of cryptic female choice (Birkhead and Møller 1998; Simmons 2001) can all influence variation in paternity share and generate deviations from the predictions of our null model assuming of equality of mating. Such processes may themselves erode the predictive power of SCI in determining a male’s paternity share, as the number of males that inseminate a female becomes less important in determining male fertilisation success relative to other factors, e.g. the timing of insemination or ejaculate traits (Parker and Pizzari 2010). This may in turn reduce the predictive power of SCIC. Such cases will themselves provide interesting empirical studies in understanding how the pattern of female multiple mating determines the operation of sexual selection. A particularly relevant example arises when individual partners copulate with each other multiple times (i.e. edges between males and females nodes are weighted by the number of copulations). Remating between male and female pairs may reflect postcopulatory selection on males and function as a trait that allows a male to defend his paternity by increasing the relative representation of his ejaculates versus competitors (Shuster and Wade 2003). In such situations, the calculation of a male’s SCI can be extended to include multiple copulations between individual males and females. Using this weighted approach, a male’s exclusivity with a female partner is determined not by the number of competitor males but instead by the relative representation of his ejaculates (Shuster and Wade 2003). This weighted version of male SCI is therefore shaped by both the remating rate of the focal male and the mating rates of competitor males, i.e. a low weighted SCI can be the result of both high focal male remating rate and low remating rates of other competitor males. Weighted and unweighted versions of SCIC can be compared to explore how patterns of remating accentuate or diminish the relationship between male mating success and the intensity of sperm competition.

The simulation approach used in this work has provided hypothetical conditions in which the researcher has detailed and complete knowledge of the mating patterns of a population However, in many empirical studies, researchers may have less detailed knowledge, e.g. when mating success is inferred from genetic parentage assignment with little or no behavioural data (Collet et al. 2014; Taylor et al. 2014). Using genetic data alone may result in overestimates of variance in male mating success and Bateman gradients (Collet et al. 2014). With reference to the estimation of assortative mating structure, we expect that using genetic parentage as a means of determining male–female mating networks may also bias results. When behavioural data are not available, genetically determined mating networks may not accurately characterise the distribution of sperm competitive environments across males and instead may better represent the results of sperm competition rather than the competitive landscape, potentially underestimating the assortativity of a given mating network. This problem may be exacerbated when female clutch size is small (Collet et al. 2014). An extreme example comes from cases where females only lay one egg. In this case, using genetic parentage assignment removes completely the potential for female polyandry and any level of mating structure; however, the distribution of reproductive success across males may strongly depend on female polyandry and mating structure in such ‘winner fertilise all’ cases. A similar issue relates to the accuracy of behavioural data when available. Missing data may result in an underestimation of polyandry and bias estimates of mating network structure, which may be particularly strong when males of a given mating success are associated with a particular reproductive tactic that is consistently not recorded (e.g. alternative reproductive tactics; sneak mating). We therefore advocate that use of such methods requires both genetic and detailed behavioural data on copulations or genetic parentage assignment only in those cases where genetic data are strongly representative of behavioural mating patterns. Although these conditions may appear restrictive, there is great potential to explore these methods in experimental groups where behavioural data are more readily available (e.g. Collet et al. 2012; Pélissié et al. 2014) and in some natural populations of both vertebrates and invertebrates, where detailed behavioural data are becoming available (e.g. Preston et al. 2005; Rodríguez-Muñoz et al. 2010).

Finally, although this study has focused largely on the utility of assortment measures in studies on male Bateman gradients, assortment measures such as SCIC may also have useful applications for studies focusing on female reproduction. Consider, for example, populations where males are sperm limited and so those male that mate many times may fail to deliver sufficient sperm per copulation to fertilise all the ova of a female (Warner et al. 1995; Wedell et al. 2002). Patterns of positive assortative mating may then weaken female Bateman gradients, because increasing mating success is associated with fewer sperm delivered per copulation. Whereas in negatively assorted mating network, female Bateman gradients would be strengthened because females with few mates suffer reduced fertility as their partners tend to be the most promiscuous, and so potentially the most sperm depleted.

This work merges network theory from both social sciences and ecological literature to test the utility of a variety of measures in quantifying the relationship between male mating success and the mating success of their female mating partners, for use in the study of sexual selection and sperm competition. Overall, our results lead us to suggest that the measure of SCIC is the most promising approach as it is both logically consistent with the relationship between male mating success and the mating success of their partners, is not strongly confounded by variation in population parameters and has a clear relationship to sexual selection theory.

## Electronic supplementary material

ESM 1 (TXT 3 kb)
